# Healthy lifestyle and HPV infection risk: results from a cross-sectional study

**DOI:** 10.3389/fpubh.2024.1421636

**Published:** 2024-10-02

**Authors:** Xi Zhao, Yue Wu, Huangyu Hu

**Affiliations:** ^1^Nanchong Central Hospital, Capital Medical University Affiliated Beijing Anzhen Hospital Nanchong Hospital, The Second Clinical Medical College, North Sichuan Medical College, Nanchong, China; ^2^Sichuan University West China Second University Hospital, Chengdu, China; ^3^Acupuncture School of Hospital of Chengdu University of Traditional Chinese Medicine, Chengdu, China

**Keywords:** dietary quality, HPV, healthy eating index, NHANES, physical activity

## Abstract

**Background:**

Human Papillomavirus (HPV) infection constitutes a significant global public health challenge despite the widespread implementation of vaccination programs, with infection rates persistently high. Recent studies suggest that lifestyle factors including diet quality (DQ) and physical activity (PA) could play a pivotal role in the mitigation of HPV infections. This investigation explored the influence of DQ, PA, and a healthy lifestyle on the incidence of HPV infection in adult women.

**Methods:**

Data from 5,308 women aged 20–59 from the National Health and Nutrition Examination Survey (NHANES) 2007–2016. DQ and PA were measured using the Healthy Eating Index (HEI-2015) and the Global Physical Activity Questionnaire (GPAQ), respectively, creating four lifestyle groups based on how well they matched dietary and activity guidelines. The associations between key factors and HPV infection were explored using multivariate logistic regression, trend tests, and interaction tests.

**Results:**

Fully adjusted multivariable logistic regression models revealed an inverse association between the risk of HPV infection and higher levels of PA (OR = 0.914; 95% CI: 0.854–0.979) as well as DQ (OR = 0.993; 95% CI: 0.989–0.998). Individuals in the highest tertile of DQ displayed a reduced risk of HPV infection relative to those in the lowest tertile (OR = 0.846; 95% CI: 0.726–0.986). Belonging to the third quintile of PA was linked to a lower risk of HPV infection than the lowest quintile (OR = 0.823; 95% CI: 0.681–0.995). Subgroups adhering to a healthy DQ were linked to a lower risk of HPV infection irrespective of PA meeting guideline recommendations.

**Conclusion:**

Our findings underscore the importance of a healthy diet in conjunction with appropriate PA in preventing HPV infection, offering new insights for public health policies and interventions.

## 1 Introduction

Human papillomavirus (HPV) is a DNA virus associated with infections of the skin and mucous membranes. To date, over 200 subtypes have been identified (1). In the United States, HPV infection is the most common sexually transmitted infection. The Centers for Disease Control and Prevention has reported that 79 million Americans are currently infected with HPV, with an additional 14 million individuals contracting the infection annually (2). Among women aged 18 to 59, the prevalence of genital HPV infection is estimated to be up to 43% (3). The health burden attributed to HPV infections in women is significantly higher compared to men (4).

Despite the increasing coverage of HPV vaccinations, many individuals remain at risk for HPV infection. It is imperative to identify and modify factors that can mitigate this risk. Research has highlighted the crucial roles of dietary quality (DQ) and physical activity (PA) in the prevention of HPV infection. Studies suggest that adhering to healthy dietary patterns, such as the Mediterranean diet, can offer protective benefits against high-risk HPV infections (5). Moreover, a high consumption of vegetables, legumes, and fruits is associated with a lower incidence of vaginal HPV infections among American women (6). PA, defined as any movement that expends energy through skeletal muscle work, has been shown to reduce the risk of at least 25 chronic diseases by 20–30% (7, 8). Evidence also suggests a significant association between vigorous-intensity PA and a reduced risk of HPV infection (9).

Nonetheless, there are several issues and limitations with the existing body of research. For instance, studies examining the relationship between DQ and HPV infection have not accounted for confounders such as HPV vaccination status (6). Research on the association between PA and HPV infection is scarce, lacking consensus and depth. Additionally, the focus has been predominantly on the individual effects of either DQ or PA, with no studies comprehensively investigating the synergistic impact of both. To address these gaps, we aim to utilize the National Health and Nutrition Examination Survey (NHANES) data to holistically assess the influence of both DQ and PA on the risk of HPV infection.

## 2 Methods

### 2.1 Study populations

The NHANES database served as the source for all collected data. The study has received approval from the U.S. ethics committee, with informed consent obtained from all participants. It represents a population-based cross-sectional survey.

**Figure 1:**
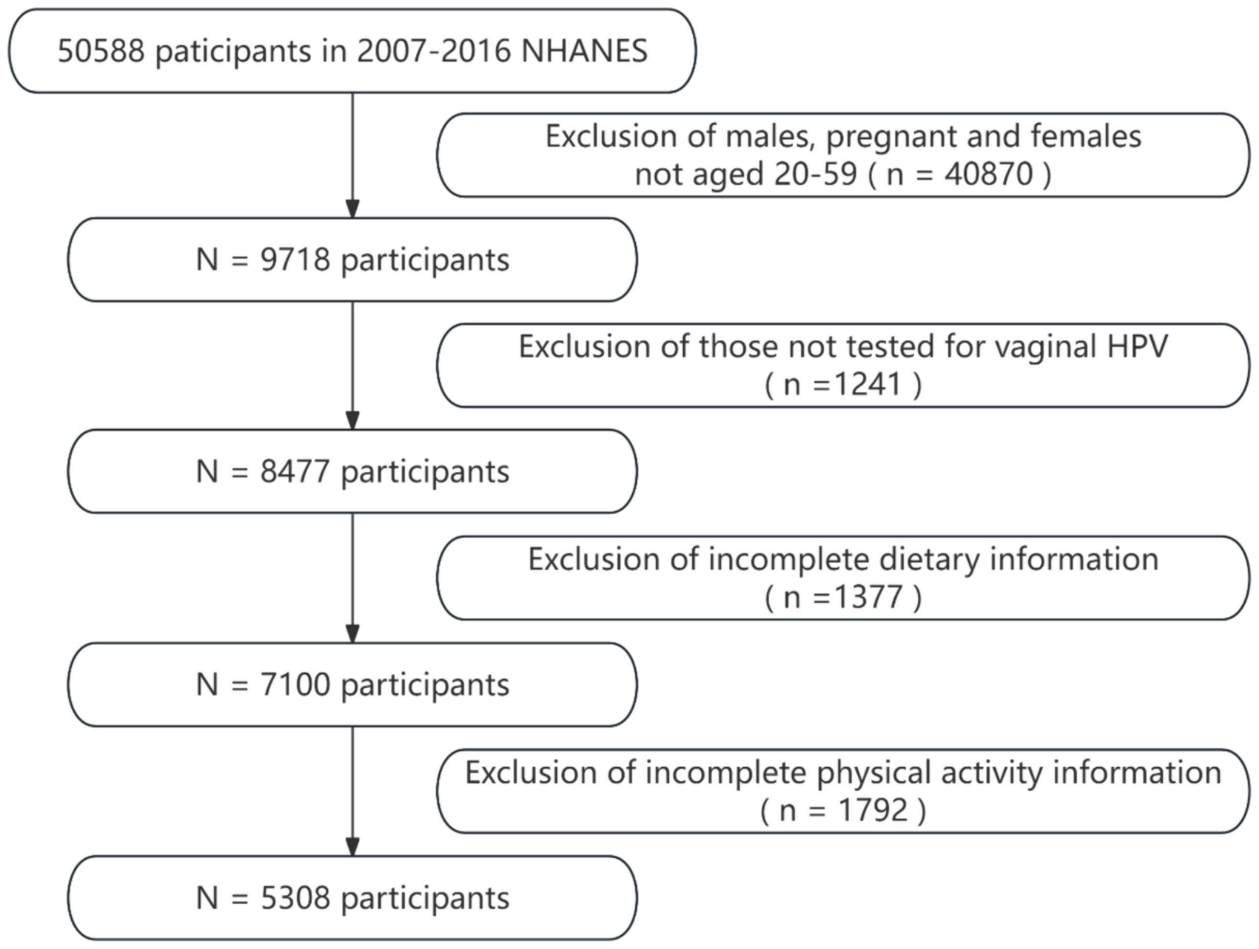
Flowchart of participants selection.

Data were chosen from five NHANES biennial cycles (2007–2008, 2009–2010, 2011–2012, 2013–2014, and 2015–2016), selected specifically for the availability of pertinent data, such as HPV vaccination, only found within these periods. The inclusion criteria for participants were: (1) being female, aged 20 to 59; (2) having undergone vaginal HPV testing; and (3) possessing complete dietary and PA information. Women who were pregnant or unsure about their pregnancy were excluded. These criteria were established to assure the data’s completeness and relevance. In the end, 5,308 women were incorporated into the study’s dataset. The process of data selection is depicted in Figure 1.

### 2.2 Dietary quality

The Healthy Eating Index (HEI-2015) serves as a metric to assess the quality of an individual’s diet by evaluating how well a variety of foods align with the “2015–2020 Dietary Guidelines for Americans” (10). This index is comprised of 13 components categorized into two major groups: Adequate Intake Components, which include fruits, vegetables, whole grains, dairy, protein foods (including seafood and plant-based proteins), and essential fatty acids that should be consumed in ample amounts; and Moderate Intake Components, which involve intake of refined grains, sodium, added sugars, and saturated fats in moderation. Dietary data is obtained through dual 24-h dietary recalls of all foods and beverages consumed on the preceding day, and HEI-2015 scores are calculated using the United States Department of Agriculture Food Patterns Equivalents Database. Scores range from 0 to 100, with higher values indicating superior DQ. If a participant’s mean score across 2 days meets or surpasses the upper tertile (T3), their dietary habits are deemed to be of healthy diet, consistent with the guidelines’ recommendations for healthy eating (11).

### 2.3 Physical activity

The Global Physical Activity Questionnaire (GPAQ), endorsed by the World Health Organization, serves as an instrumental tool for gauging the PA levels among populations worldwide. Utilized within the NHANES, the GPAQ facilitates the collection of data regarding an individual’s engagement in physical activities across various sectors, including work, transportation, and leisure, over the preceding week. This methodology allows for a comprehensive assessment of PA patterns, contributing to the global monitoring of health and fitness trends (12).

In the comprehensive evaluation of PA, the total is commonly expressed as the weekly cumulative number of Metabolic Equivalent (MET) minutes, capturing the aggregate time spent across all activity types. The calculation of MET minutes for each activity leverages standard MET values provided by NHANES, subsequently determined by the activity’s nature, frequency, and duration. The formula used is: PA (MET-minutes/week) = MET value × weekly frequency of each activity × duration. To ascertain if the participants’ activity levels reach the recommended threshold by the U.S. Department of Health and Human Services Physical Activity Guidelines for Americans, their weekly accumulation of at least 600 MET minutes is evaluated (13). This benchmark equates to 150 min of moderate-intensity or 75 min of high-intensity PA per week. Following this standard, participants’ physical activities are categorized as either meeting (≥600 MET minutes per week) or not meeting (<600 MET minutes per week) the guidelines. For data processing and analysis simplicity, MET minutes are converted to MET hours by dividing the total MET minutes by 60, facilitating a more straightforward unit conversion.

### 2.4 Lifestyle subgroups

Building upon the grouping methods utilized in previous studies, and considering both the DQ and the adherence to recommended PA standards of the participants, this study divides them into four distinct groups (11, 14). These are: Group 1, adhering to the PA guidelines and maintaining a healthy diet; Group 2, adhering to the PA guidelines but consuming an unhealthy diet; Group 3, not adhering to the PA guidelines yet maintaining a healthy diet; and Group 4, neither adhering to the PA guidelines nor maintaining a healthy diet. This classification approach facilitates a detailed investigation into how different lifestyle combinations influence the risk of HPV infection.

### 2.5 HPV infection status

Within the scope of this study, the dataset focuses on individuals aged 20 to 59 years, all of whom are registered within public databases. To safeguard the precision of HPV infection data, the Roche Linear Array HPV Genotyping Test has been employed as the primary methodological approach. This choice is predicated on its capability to effectively identify and detect a broad spectrum of HPV subtypes, encompassing, yet not limited to, types 6, 11, 16, 18, through to IS39. According to our criteria, a positive result for any HPV subtype is deemed indicative of an HPV infection.

### 2.6 Covariates

In the context of this study, covariates were selected based on insights gleaned from existing literature and clinical practice. The selected covariates included age, race, poverty income ratio (PIR), marital status, education level, smoking history, alcohol consumption, age of sexual debut (<18 or ≥ 18), number of sexual partners (0–1, 2–5 or > 5), and HPV vaccination (yes or no). The data pertaining to these covariates were sourced from the demographic, examination, reproductive health, and sexual behavior questionnaires of the NHANES database. Based on alcohol consumption, drinking history was categorized as never (< 12 lifetime drinks), former (≥ 12 drinks in 1 year but no drinks in the last year or ≥ 12 lifetime drinks but no drinks in the last year), mild (≤ 1 daily drinks in the last 12 months), moderate (2 daily drinks in the last 12 months), or heavy (≥ 3 daily drinks in the last 12 months). Smoking history was classified as never (< 100 lifetime cigarettes), former (> 100 lifetime cigarettes but not a current smoker), or current.

### 2.7 Statistical analysis

The approach to data analysis in this investigation was conducted in strict compliance with the methodological guidelines set forth by the NHANES, utilizing a weighted scheme. The rationale behind employing a weighted analysis lies in its ability to extend the outcomes derived from the sample to the broader population of non-institutionalized U.S. residents, thus improving the universality and representativeness of the study’s conclusions (15).

In describing the baseline data for this study, continuous variables that adhere to a normal distribution are denoted by their mean values and corresponding standard errors (SE). In contrast, those deviating from a normal distribution are depicted using median values and the interquartile range. For the analysis of continuous variables that follow a normal distribution, univariate analysis of variance is utilized, offering a means to assess variance within and between groups. On the other hand, for continuous variables that do not follow a normal distribution, the Kruskal-Wallis test is employed, providing a non-parametric alternative for statistical evaluation. Categorical variables are examined through the chi-square test and are presented as percentages, facilitating a straightforward interpretation of the data in terms of frequency distribution.

This study employed a multivariate logistic regression model to investigate the associations between PA, DQ, lifestyle factors, and HPV infection. To gain an in-depth understanding of the correlations across different demographics, the study conducted stratified multivariable regression analysis and assessed the presence of significant differences among various subgroups by performing interaction tests. In all statistical analyses, a *p*-value of less than 0.05 was adopted as the threshold for statistical significance. The complete data analysis was carried out using the R statistical software (version 4.2.3, accessible at http://www.r-project.org). Employing this analytical approach allowed for a comprehensive evaluation of the relationship between body weight and HPV infection, while considering numerous potential confounding factors.

## 3 Results

### 3.1 Baseline characteristics of the study population

Table 1 presents the clinical baseline characteristics of participants, stratified by their lifestyle choices. This research encompassed 5,308 individuals, with an age-adjusted average of 39.65 years (SE 0.34), among whom 39.45% were categorized as infected with HPV. Participants maintaining a healthy diet coupled with regular PA had an HPV infection prevalence of 36.74%, an average HEI score of 70.14 (SE 0.31), and engaged in moderate to vigorous PA amounting to 38 (range 22 to 74) MET-hours weekly. By contrast, those lacking both in diet and exercise had an infection rate of 43.21%, an average HEI score of 46.93 (SE 0.36), and reported only 6 (range 4 to 8) weekly hours of similar intensity PA. Statistically significant disparities were observed among the four lifestyle categories concerning age, race, PIR, marital status, education level, alcohol consumption, smoking history, HEI score, PA, age of sexual debut, HPV vaccination, and HPV infection status (*p*-values all <0.05). The number of sexual partners, however, did not show significant variation across groups (*p* value >0.05).

**Table 1:** Baseline characteristics of study population according to lifestyle.

Variable	Total	Lifestyle categories				*P* value
		Group 1^a^	Group 2^b^	Group 3^c^	Group 4^d^	
Age (year), Mean (S.E)	39.65 (0.34)	40.26 (0.50)	38.04 (0.45)	45.35 (0.93)	41.01 (0.50)	< 0.001*
HEI, Mean (S.E)	55.31 (0.39)	46.93 (0.36)	47.25 (0.26)	70.12 (0.74)	70.14 (0.31)	< 0.001*
PA (MET-h/week),	30 (12,72)	6 (4, 8)	44 (21,100)	6 (4, 8)	38 (22, 74)	< 0.001*
PIR, Median (S.E)	2.97 (1.39,5.00)	2.70 (1.26,4.80)	2.42 (1.22,4.40)	3.51 (1.43,5.00)	3.87 (1.96,5.00)	< 0.001*
Race, *n* (%)						< 0.001*
Black	1,113 (11.94)	196 (14.49)	616 (13.60)	61 (9.66)	240 (8.34)	
Mexican	817 (8.82)	105 (7.37)	424 (9.75)	69 (12.82)	219 (7.25)	
Other	1,157 (13.74)	152 (12.71)	529 (12.79)	110 (24.09)	366 (13.91)	
White	2,221 (65.50)	324 (65.43)	1,191 (63.86)	98 (53.42)	608 (70.50)	
Marital status, *n* (%)						0.002*
Divorced/separated/widowed	936 (15.67)	140 (16.95)	506 (16.61)	64 (15.73)	226 (13.46)	
Married/living with a partner	3,092 (62.92)	459 (63.22)	1,510 (59.31)	221 (71.01)	902 (67.34)	
Never married	1,280 (21.41)	178 (19.83)	744 (24.09)	53 (13.26)	305 (19.20)	
Education level, *n* (%)						< 0.001*
<High school	936 (12.58)	166 (16.38)	512 (14.05)	58 (11.78)	200 (8.42)	
>High school	3,340 (68.92)	429 (56.62)	1,644 (65.01)	223 (76.04)	1,044 (80.15)	
High school	1,032 (18.50)	182 (27.01)	604 (20.94)	57 (12.18)	189 (11.43)	
Age of first sex, *n* (%)						< 0.001*
<18	2,903 (55.44)	472 (59.88)	1,682 (60.76)	124 (43.04)	625 (46.65)	
≥18	2,405 (44.56)	305 (40.12)	1,078 (39.24)	214 (56.96)	808 (53.36)	
Alcohol consumption, *n* (%)						< 0.001*
Former	615 (10.84)	104 (11.10)	341 (12.44)	33 (10.88)	137 (8.01)	
Heavy	1,211 (23.67)	174 (24.71)	723 (25.92)	47 (19.11)	267 (20.22)	
Mild	1,384 (27.66)	195 (27.48)	659 (24.84)	97 (30.88)	433 (31.87)	
Moderate	1,204 (25.13)	168 (23.40)	614 (23.96)	68 (20.19)	354 (28.85)	
Never	894 (12.71)	136 (13.31)	423 (12.84)	93 (18.94)	242 (11.05)	
Smoking history, *n* (%)						< 0.001*
Former	799 (17.60)	122 (16.19)	389 (16.15)	46 (17.71)	242 (20.71)	
Never	3,461 (62.38)	472 (58.54)	1,659 (57.81)	263 (75.09)	1,067 (69.55)	
Now	1,048 (20.02)	183 (25.27)	712 (26.05)	29 (7.20)	124 (9.74)	
Sexual partners, *n* (%)						0.055
0–1	1,110 (18.60)	154 (16.62)	487 (17.53)	112 (27.22)	357 (19.75)	
2–5	2038 (37.88)	291 (40.62)	1,057 (36.53)	132 (38.16)	558 (38.76)	
>5	2,160 (43.53)	332 (42.77)	1,216 (45.94)	94 (34.63)	518 (41.49)	
HPV vaccine *n* (%)						0.004*
No	4,829 (90.35)	726 (93.91)	2,483 (88.98)	318 (95.84)	1,302 (89.92)	
Yes	479 (9.65)	51 (6.09)	277 (11.02)	20 (4.17)	131 (10.08)	
HPV infection status, *n* (%)						< 0.001*
No	3,059 (60.55)	417 (56.79)	1,500 (58.32)	232 (75.95)	910 (63.27)	
Yes	2,249 (39.45)	360 (43.21)	1,260 (41.68)	106 (24.05)	523 (36.74)	

^aGroup 1: unhealthy diet and inactive individuals.^

^bGroup 2: unhealthy diet but active individuals.^

^cGroup 3: healthy diet but inactive individuals.^

^dGroup 4: healthy diet and active individuals.^

PA, physical activity; HEI, healthy eating index; PIR, poverty income ratio; *P < 0.05.

### 3.2 Association between DQ or PA and HPV infection

We assessed the association between individual DQ and PA with HPV infection using weighted multivariate logistic regression (Table 2). In all three models, we consistently observed that a higher DQ was associated with a decreased likelihood of HPV infection (OR = 0.993; 95%CI: 0.989–0.998), indicating that each unit increase in DQ corresponded to a 0.7% reduction in the risk of HPV infection. For sensitivity analysis, DQ was reclassified from a continuous variable into a categorical variable (tertiles). In the fully adjusted model, participants in the highest tertile of DQ experienced a statistically significant 15.4% reduction in the risk of HPV infection compared to those with the lowest DQ. The test for trend was significant, yielding a *p*-value of 0.03, which implies that the observed trend change is meaningful.

**Table 2:** Association between DQ, PA, lifestyle and HPV infection status.

Exposure	Model 1^a^ OR, 95% CI	Model 2^b^ OR, 95% CI	Model 3^c^ OR, 95% CI
Lifestyle			
**Lifestyle category**			
Group 1^d^	Ref	Ref	Ref
Group 2^e^	1.002 (0.858,1.170)	1.005 (0.858,1.177)	0.918 (0.775,1.088)
Group 3^f^	0.559 (0.432,0.721)	0.624 (0.480,0.809)	0.756 (0.571,0.996)
Group 4^g^	0.680 (0.571,0.809)	0.733 (0.614,0.875)	0.809 (0.668,0.980)
P for trend	<0.001*	<0.001*	0.016*
DQ			
DQ continuous	0.982 (0.978,0.986)	0.985 (0.981,0.990)	0.993 (0.989,0.998)
DQ tertile			
T1 [19.9,48.2]	Ref	Ref	Ref
T2 [48.2,60.7]	0.837 (0.733,0.955)	0.872 (0.762,0.999)	0.990 (0.856,1.146)
T3 [60.7,94.4]	0.596 (0.521,0.682)	0.659 (0.574,0.758)	0.846 (0.726,0.986)
*P* for trend	<0.001*	<0.001*	0.03
PA			
PA Continuous	0.829 (0.783,0.878)	0.832 (0.784,0.883)	0.914 (0.854, 0.979)
PA quintile			
Q1[0.6,9]	Ref	Ref	Ref
Q2 [9,20.6]	0.975 (0.820,1.158)	0.985 (0.826,1.173)	0.988 (0.818,1.194)
Q3 [20.6,42]	0.830 (0.698,0.987)	0.828 (0.694,0.987)	0.823 (0.681,0.995)
Q4 [42,94]	1.077 (0.907,1.279)	1.063 (0.892,1.267)	0.986 (0.816,1.191)
Q5 [94,672]	1.240 (1.045,1.470)	1.182 (0.993,1.407)	0.969 (0.803,1.169)
*P* for trend	<0.001*	0.003*	0.843

^aModel 1: no covariates were adjusted.^

^bModel 2: adjusted for age and race.^

^cModel 3: adjusted for age, race, PIR, marital status, education level, alcohol consumption, history of smoking, age of first sex, sexual partners and HPV vaccine.^

^dGroup 1: unhealthy diet and inactive individuals.^

^eGroup 2: unhealthy diet but active individuals.^

^fGroup 3: healthy diet but inactive individuals.^

^gGroup 4: healthy diet and active individuals.^

DQ, diet quality; PA, physical activity; *P<0.05.

Similarly, in Model 3, a negative correlation was observed between PA and HPV infection (OR = 0.914; 95%CI: 0.854–0.979), indicating that each additional hour of moderate to intense PA per week was associated with a 0.96% reduction in the risk of HPV infection. PA was subsequently reclassified from a continuous variable to quintile categories. It was found that across all three models, the risk of HPV infection decreased when comparing Q3 to Q1. However, in Model 1, an increased risk of HPV infection was noted when Q5 was compared to Q1. This relationship was not evident in the covariate-adjusted Models 2 and 3.

### 3.3 Association between lifestyle HPV infection

The association of various lifestyle combinations with HPV infection is depicted in Table 2. In Model 3, a negative correlation was observed between both Group 3 and Group 4 and HPV infection, relative to Group 1. The effect values for these groups were OR = 0.756; 95% CI: 0.571–0.996 and OR = 0.809; 95% CI: 0.668–0.980, respectively.

### 3.4 Subgroup analysis

To further assess the consistency of the lifestyle-HPV infection relationship across populations, we performed subgroup analyses stratified by age, complemented by an interaction test (Table 3). In the unadjusted model, a 68.6% decrease in HPV infection risk was observed in the 20–29 years age group for group 3 as compared to group 1 (OR = 0.314; 95% CI, 0.114–0.860), and a 53.6% reduction in infection risk was noted for group 3 within the 50–59 years age group (OR = 0.464; 95% CI, 0.221–0.973). However, the interaction test results indicated no significant differences between groups (P for interaction = 0.694). In the fully adjusted model, group 2 exhibited a 48.4% lower risk of HPV infection relative to group 1 (OR = 0.516; 95% CI, 0.316–0.842), and the interaction test yielded no statistical difference (P for interaction = 0.745).

**Table 3:** Subgroup analysis for the association between lifestyle and HPV infection status.

Character	Group 1^a^	Group 2^b^	Group 3^c^	Group 4^d^	*P* for interaction
Unadjusted					
Age					0.694
20–29	Ref	0.703 (0.475,1.041)	0.314 (0.114,0.860)	0.745 (0.498,1.116)	
30–39	Ref	1.028 (0.599,1.765)	0.413 (0.154,1.105)	0.795 (0.442,1.429)	
40–49	Ref	1.093 (0.730,1.638)	0.570 (0.303,1.073)	0.716 (0.450,1.138)	
50–59	Ref	0.821 (0.525,1.282)	0.464 (0.221,0.973)	0.808 (0.457,1.431)	
Adjusted ^e^					
Age					0.745
20–29	Ref	0.516 (0.316,0.842)	0.407 (0.128,1.299)	0.592 (0.342,1.022)	
30–39	Ref	1.128 (0.594,2.139)	0.611 (0.198,1.890)	1.182 (0.610,2.294)	
40–49	Ref	1.019 (0.658,1.576)	0.729 (0.345,1.537)	0.897 (0.540,1.490)	
50–59	Ref	0.724 (0.443,1.182)	0.461 (0.207,1.026)	1.006 (0.550,1.841)	

^aGroup 1: unhealthy diet and inactive individuals.^

^bGroup 2: unhealthy diet but active individuals.^

^cGroup 3: healthy diet but inactive individuals.^

^dGroup 4: healthy diet and active individuals.^

^eAdjusted model: adjusted for age, race, PIR, marital status, education level, alcohol consumption, history of smoking, age of first sex, sexual partners and HPV vaccine.^

## 4 Discussion

In this study, the impacts of DQ, PA, and lifestyle on HPV infection were examined. A negative association was observed between DQ, PA, and HPV infection. Among the lifestyle groups, respondents with superior DQ had a lower likelihood of HPV infection compared to those with inferior DQ and inadequate PA, irrespective of their adherence to PA guidelines. These results carry significant implications for the formulation of public health interventions and offer potential strategies for lifestyle modification to mitigate HPV infection.

The HEI is an important tool for evaluating DQ, and high HEI scores typically indicate adequate intake of nutrients such as fruits, vegetables, and whole grains, as well as a low intake of refined grains, sodium, added sugars, and saturated fats, which are generally associated with better health outcomes (16). In our study, higher HEI scores were inversely associated with a lower risk of HPV infection, a result that remained consistent irrespective of whether HEI was analyzed as a continuous or a categorical variable. Our results are in alignment with previous research (6), and by incorporating HPV vaccination as a crucial control variable, we have augmented the precision of our study. Healthy dietary patterns contribute to reinforcing the immune system, an essential factor for preventing HPV infection (17). Dietary antioxidants, abundant in foods like vegetables, fruits, and whole grains, may play an integral role in combatting HPV infection by modulating immune responses and viral replication (18). Furthermore, the intake of vitamins A, B2, E, and folic acid has been significantly associated with a decreased risk of HPV infection (19). The association between a high intake of total and whole fruits, as well as seafood/plant protein, and a lower risk of HPV infection was also corroborated in a prospective cohort study (20).

Our study determined that a modest increase in moderate to vigorous intensity PA, namely an extra hour of activity per week, correlated with a slight reduction in the risk of HPV infection. This parallels the findings of a study from China that utilized smartphones to monitor PA and determined that increased daily hours of PA could potentially reduce the risk of HPV infection (21). The presence of this association lends support to the theory that PA beneficially influences immune function, potentially aiding in the prevention of HPV infection (22). However, this relationship was not linear, evidenced by the lack of further reduction in infection risk among those with the highest levels of PA. This implies that excessive PA may not confer additional benefits and might even negate the positive effects of low-dose activity, as indicated in certain studies (23). This resonates with another study involving more than 13,000 runners which determined that escalating the dose of jogging did not further decrease long-term mortality (24). This finding highlights the potential of PA as a preventive strategy, while also illustrating the importance of maintaining an appropriate balance of activity levels to realize a preventive effect. Additionally, evidence suggests that the optimal exercise dosage for maximum health benefits varies among individuals and across different clinical outcomes (25). This further highlights the necessity to tailor recommended PA levels and the significance of considering individual differences in public health recommendations (26).

Concurrently, our analysis of lifestyle combinations reinforced the notion that a healthy diet may exert a positive effect on preventing HPV infection. We discovered that enhancing dietary habits alone, even in the absence of increased PA, reduced the risk of infection. This is at odds with the prevailing health paradigm that posits more PA as inherently beneficial. This apparent contradiction could stem from the relatively low benchmark for PA (10 MET-hours/week) set in our study, which likely included a greater number of individuals engaging in high-intensity PA. As previously indicated, excessive PA might negate the benefits derived from low-intensity activity. In our study, there is a possibility that high levels of PA detracted from the protective effects imparted by a healthy diet, to a certain degree. This intimates that excessive PA might influence the body’s response to a healthy diet, a hypothesis that necessitates additional research and scrutiny.

HPV consists of over 200 strains, with high-risk types like HPV 16 and 18 being linked to cancers such as cervical and oropharyngeal cancers, while low-risk types like HPV 6 and 11 cause benign conditions such as genital warts (1, 2). Preventing HPV infections is crucial for reducing the burden of these associated diseases. Our study underscores the importance of lifestyle factors in mitigating HPV infection risk, revealing that higher DQ and increased PA are inversely associated with HPV infection. Additionally, promoting public awareness about the benefits of these healthy behaviors is essential. Educational campaigns should emphasize the role of a balanced diet and regular exercise in preventing HPV infections and related cancers. Furthermore, integrating HPV vaccination into routine healthcare for adolescents and young adults is crucial, as it has been proven to significantly reduce the prevalence of high-risk HPV strains (27). Smoking cessation programs are also critical, as smoking is a known cofactor in the progression of HPV to cervical cancer (28). By combining these strategies—nutritional guidance, physical activity promotion, vaccination, and smoking cessation—public health initiatives can create a comprehensive approach to mitigate HPV infection risks. This multifaceted strategy not only addresses the direct prevention of HPV but also fosters overall well-being, providing robust insights for public health policies and interventions aimed at reducing the global burden of HPV-related diseases.

The strength of this study is rooted in the cross-sectional data derived from NHANES, encompassing 5,308 women and weighted in accordance with the NHANES guidelines. Additionally, the synergistic effects of DQ and PA on HPV infection received a thorough evaluation. However, the study also has its inherent limitations. Owing to the cross-sectional design of this study, establishing causality was not possible. The evaluation of DQ and PA levels relied predominantly on participants’ self-reported data, potentially subject to recall bias and social desirability bias. Observations stemmed predominantly from women aged 20 to 59 years, potentially limiting the generalizability of our findings to other age demographics.

## 5 Conclusion

In this study, the influences of individual DQ, PA, and various lifestyle patterns on the risk of HPV infection in adult women were systematically investigated. Upon analyzing data from 5,308 women aged 20 to 59 years, it was found that high-quality dietary habits and moderate PA are significantly associated with a reduced risk of HPV infection. This discovery underscores the pivotal role that a healthy lifestyle plays in mitigating HPV infection and posits that the promotion of a nutritious diet and enhanced PA could be efficacious in diminishing HPV risk. Considering the associations between HPV infection and numerous severe health issues, such as cervical cancer, this study offers important insights into public health interventions.

## Data Availability

The datasets presented in this study can be found in online repositories. The names of the repository/repositories and accession number (s) can be found below: the data for this study are already publicly available through the National Center for Health Statistics (NCHS), National Health and Nutrition Examination Survey (NHANES) website: https://www.cdc.gov/nchs/nhanes/about_nhanes.htm.
